# Microgravity‐induced changes in skeletal muscle and possible countermeasures: What we can learn from bed rest and human space studies

**DOI:** 10.1113/EP092345

**Published:** 2025-03-17

**Authors:** Alessandra Bosutti, Bergita Ganse, Nicola A. Maffiuletti, Rob C. I. Wüst, Gustav J. Strijkers, Andy Sanderson, Hans Degens

**Affiliations:** ^1^ Department of Life Sciences University of Trieste Trieste Italy; ^2^ Departments and Institutes of Surgery Saarland University Homburg Germany; ^3^ Human Performance Lab Schulthess Clinic Zurich Switzerland; ^4^ Department of Human Movement Sciences, Faculty of Behavioural and Movement Sciences, Amsterdam Movement Sciences Vrije Universiteit Amsterdam Amsterdam The Netherlands; ^5^ Department of Biomedical Engineering and Physics, Amsterdam UMC University of Amsterdam Amsterdam The Netherlands; ^6^ Department of Sport and Exercise Sciences, Institute of Sport Manchester Metropolitan University Manchester UK; ^7^ Department Life Sciences Manchester Metropolitan University Manchester UK; ^8^ Institute of Sport Science and Innovations Lithuanian Sports University Kaunas Lithuania

**Keywords:** artificial gravity, astronauts, bed rest, ISS, microgravity, muscle atrophy, NMES, spaceflight

## Abstract

Despite exercise countermeasures to sustain health and performance in spaceflight, complete maintenance of muscle mass and functions in microgravity is still not possible for most astronauts. The principal cause of the limited effectiveness of existing exercise countermeasures is the difficulty in achieving full loading forces in space. The implementation of countermeasures which require small devices and simulate Earth‐like loading forces to maintain muscle mass, strength and endurance is therefore highly desirable. At present, the cellular mechanisms that induce muscle atrophy in weightlessness are not yet fully known; a better understanding of how skeletal muscle cells adapt to microgravity will help in designing more effective countermeasures to sustain the health and operational capacity of the crew during long‐ and short‐duration missions. The 6° head‐down‐tilt bed rest is a powerful ground‐based analogue platform to simulate and study the physiological effects of spaceflight on the human body, and test the effectiveness of countermeasures before they are potentially applied in space. The aims of this narrative review are therefore to provide an overview of (i) the main mechanisms underlining muscle atrophy learnt from space and bed rest studies, (ii) the currently available countermeasures, and (iii) potential suitable countermeasures – such as neuromuscular electrical stimulation that is delivered with light and small portable units – to attenuate muscle wasting in astronauts during spaceflight.

## Abstract (highlights)

- **What is the topic of this review?**
  This review provides an overview of the main mechanisms underlying muscle deconditioning in microgravity environments and potential exercise countermeasures to attenuate muscle wasting in space.
- **What advances does it highlight?**
  Physiological mechanisms activated by neuromuscular electrical stimulation to attenuate muscle wasting in space.

## 1 INTRODUCTION

Spaceflight induces significant changes in the human body, such as muscle atrophy, cardiovascular deconditioning and alterations in sensorimotor function (Williams et al., 2009). In addition to the microgravity‐induced loss of muscle mass, gravitational unloading may also be accompanied by increased muscle fatiguability (Koryak, 2020). These alterations, while mostly reversible, not only contribute to the rapid decline in exercise capacity, but may also impair crew operational proficiency during long‐ and even short‐duration missions, and particularly cause problems on return to Earth. In addition, metabolic alterations associated with muscle atrophy, such as a decrease in insulin sensitivity, may have a further negative impact on astronaut health (Tobin et al., 2002). During future missions, astronauts will be exposed to even longer periods of microgravity or reduced gravity environments as on the Moon or Mars, which may exacerbate the ability to perform work and activities of daily life. Therefore, the application of countermeasures capable of preventing microgravity‐induced body deconditioning is essential (Petersen et al., 2016; Williams et al., 2009).

To maintain muscle mass and optimal operational capacity in space, astronauts currently adhere to a daily time‐consuming exercise training programme (Petersen et al., 2016). Yet, research on the physiological and molecular impacts of existing and novel countermeasures while in space is sparse, mainly because of limited access to the International Space Station (ISS) astronauts and cosmonauts for research, caused by the small number of crew members’ permission and the constraints of crew research time available during spaceflight. To overcome these limitations, 6° head‐down‐tilt (HDT) bed rest has been used as a model for spaceflight to study (i) physiological and molecular adaptations to the absence or reduction of the gravity vector and (ii) the effectiveness of new countermeasures before their potential application in space (Pavy‐Le Traon et al., 2007).

The development of countermeasures is becoming a pressing issue as in the future missions with durations of over a year will likely become more frequent. Further, habitats established off‐planet will most likely impose strict limitations in hardware mass and volume, thus requiring lightweight and small countermeasure payloads. Amongst these, the exposure to artificial gravity (AG) has recently been studied in detail. In addition, neuromuscular electrical stimulation (NMES) is a promising training modality that not only has been shown to provide a range of beneficial effects for patients during/after a period of disuse due to surgery, injury or a pathology and for healthy subjects undergoing short periods of ground‐based models of deconditioning (Dirks et al., 2014; Zange et al., 2017), including bed rest (Hansen et al., 2024: Reidy et al., 2017), but also fulfils the requirements of being a small and lightweight type of ‘exercise equipment’ in future space missions.

In this review, we will first provide an overview of microgravity‐induced changes in skeletal muscle structure and function, followed by a discussion of some of the mechanisms involved in these adaptations, and end with promises of new countermeasures, with a special emphasis on NMES, to combat the microgravity‐induced decrement in muscle function.

## 2 PHYSIOLOGY OF MUSCLE ATROPHY: LESSONS FROM BED REST AND SPACE RESEARCH

### 2.1 Microgravity‐induced muscle atrophy and weakness

The main cause of microgravity‐induced muscle weakness is muscle atrophy, which is attributable to a reduction in fibre cross‐sectional area (FCSA). Muscle atrophy is more pronounced in oxidative and antigravity muscles (such as the soleus muscle) than in non‐postural mixed muscles (e.g., the vastus lateralis muscle), and occurs rapidly as illustrated by a reduced FCSA after just 11 days of spaceflight in the vastus lateralis (Edgerton et al., 1995). The atrophy after 17 days of spaceflight appeared to be more pronounced in type IIa (−26%) than in type I (−15%) fibres of the soleus muscle (Widrick et al., 1999). Prolonged spaceflight (i.e., 180 days mission) caused a considerable loss of fibre mass (−33%) mainly in the following order of degree: soleus muscle fibre type I *>* soleus muscle fibre type II *>* gastrocnemius fibre type I *>* gastrocnemius fibre type II (Fitts et al., 2010). Interestingly, Fitts et al. (2010) (Table 1) showed that crew members with the highest extent of type I fibre atrophy experienced also the greatest increase in the number of type II fibres, indicating a slow‐to‐fast fibre type transition.

**TABLE 1:** Summary of human space‐flight studies.

Space flight studies	Mission duration	Authors
Human space shuttle flights: STS‐32–34	5–10 days	Edgerton et al. (1995)
Human space shuttle mission (STS‐78)	17 days	Widrick et al. (1999)
ISS from increments 5 to 11 (2002–2005)	Range: 161–192 days	Fitts et al. (2010)
Human Spacelab Life Sciences shuttle missions SLS‐1 and SLS‐2	9.5 and 15 days’ space flight	Stein et al. (1996)
Human space flight on the MIR space station	>3 months	Stein et al. (1999)
Human space flight on the MIR space station	6 months	Zange et al. (1997)
Human space flight on the ISS from Increments 19 to 22 (2009–2012)	>6 months	Ade et al. (2017)
Human space flight on the ISS, missions in the years 2015–2019	Short (9–11 days) mission duration, and prolonged (>6 months) mission duration	Blottner et al. (2023)
Human space flight on the ISS	Short (9 days) mission duration, and prolonged (180 days) mission duration	Blottner et al. (2024)
Human space flight on the ISS (the NASA Twins Study)	1 year	Garrett‐Bakelman et al. (2019)
Human space flight on the ISS	6 months	Murgia et al. (2024), Capri et al. (2019), Rittweger et al. (2018)

ISS, International Space Station.

Like in spaceflight, also in bed rest the loss of muscle mass contributes to reduced muscle strength (Kawakami et al., 2001). The loss of muscle mass results ultimately from a net loss of proteins, caused by increased muscle protein breakdown and/or decreased muscle protein synthesis (Blottner et al., 2014; Table 2).

**TABLE 2:** Summary of bed rest studies with/without countermeasures.

Bed rest studies	Duration	Main outcome	Authors
Bed rest without countermeasure	10 days	Decreased fast fibre FCSA and muscle force in the vastus lateralis muscle	Monti et al. (2021)
Bed rest without countermeasure	14 days	Decreased muscle mass. Reduction in protein synthesis in anti‐gravity muscles with no changes in protein degradation	Ferrando et al. (1996)
Bed rest without countermeasure	20 days	Decreased muscle mass and strength in knee extensors	Kawakami et al. (2001)
Bed rest without countermeasure	35 days	Impaired muscle oxidative metabolism	Salvadego et al. (2011)
Bed rest without countermeasure	42 days	Fibre atrophy with maintained capillary density in the vastus lateralis muscle	Ferretti et al. (1997)
Bed rest with and without exercise countermeasure	60 days	Resistive and aerobic exercise countermeasure contrasted the bed rest‐induced FCSA atrophy in vastus lateralis and soleus muscle	Salanova et al. (2008)
Bed rest with diet countermeasure (cocktail agent, anti‐oxidants and anti‐inflammatory)	60 days	Nutritional supplements were protective against the bed rest‐induced muscle oxidative damage	Arc‐Chagnaud et al. (2020)
Bed rest with exercise countermeasure (RSL study with reactive jumps)	60 days	Jump countermeasure mitigated the bed rest‐induced decrements in fast and slow FCSA in soleus but not in vastus lateralis muscle	Blottner et al. (2020)
Bed rest with AG countermeasure	21 days	AG was effective in counteracting disuse‐induced muscle atrophy	Symons et al. (2009)
Bed rest with AG countermeasure	21 days	AG increased the torque–velocity relationship of the knee extensors and plantar flexors following bed rest	Caiozzo et al. (2009)
Bed rest with AG countermeasure (AGBRESA study)	60 days	Bed rest induced muscle fibre atrophy, capillary rarefaction, loss of muscle strength and oxidative capacity. AG countermeasure failed to mitigate these bed‐rest‐effects. Bed rest also caused a rapid reduction in systemic insulin sensitivity. Bed rest or AG countermeasure did not affect the unit number nor motor unit size in the abductor digiti minimi and tibialis anterior muscle	Eggelbusch et al. (2024), Hendrickse et al. (2022), Clément et al. (2022), Ganse et al. (2021), Kramer et al. (2021), Attias et al. (2020)
Bed rest with diet countermeasure (MEP‐ESA study)	21 days	Bed rest did not affect fibre size in soleus or in vastus lateralis muscles. Bed rest caused metabolic inflexibility and reduced muscle oxidative capacity. Whey protein with potassium bicarbonate diet supplementation attenuated the reduction in muscle oxidative capacity but not the shift in energy metabolism induced by bed rest	Bosutti et al. (2020), Rudwill et al. (2018), Bosutti et al. (2016), Blottner et al. (2014)
Bed rest with protein/bicarbonate supplementation and resistance vibration exercise	21 days	Marginal protective effect of countermeasure on whole body aerobic capacity and cardiovascular deconditioning	Guinet et al. (2020)
Bed rest with exercise countermeasure (flywheel resistance exercise)	84 days	Flywheel resistance exercise mitigated muscle atrophy, and partially the bed rest‐induced deregulation of ubiquitin system and energy metabolism	Fernandez‐Gonzalo et al. (2020)
Bed rest with exercise countermeasure (rowing ergometry and resistive exercise)	5 weeks	Exercise countermeasure reduced the disuse‐induced decrement in muscle volume in the quadriceps and calf muscle, and preserved knee extensor and plantar flexor strength. Bed rest did not affect fibre size and capillarization in vastus lateralis muscle	Krainski et al. (2014)
Bed rest with exercise countermeasure (supine cycle exercise)	18 days	Aerobic exercise attenuated the bed rest‐induced reduction in aerobic capacity and orthostatic tolerance	Shibata et al. (2010)
Bed rest with exercise countermeasure (resistance exercise: leg press training and plantar flexion training)	20 days	Resistance exercise preserved muscle size and function in the calf muscle	Akima et al. (2003)
Bed rest with RVE	21 days	RVE preserved muscle size, strength and energy metabolism in the vastus lateralis muscle	Kenny et al. (2017)

AG, artificial gravity; FCSA, fibre cross‐sectional area; RSL, reactive jumps in a sledge; RVE, resistive vibration exercise.

Several bed rest and spaceflight studies have shown that the primary mechanism for the loss of muscle mass in humans is the reduction of both whole‐body and skeletal muscle protein synthesis. For instance, following 14 days of bed rest there was a ∼50% reduction in protein synthesis in anti‐gravity muscles with no change in protein degradation (Ferrando et al., 1996; Table 2). Perhaps somewhat counterintuitively, during the initial days of spaceflight, there was a transient net increase, rather than a decrease, in whole‐body protein synthesis rate (Stein et al., 1996; Table 1). This rate, however, subsequently rapidly decreased (by 45%) (data collected between 88 and 186 days of mission) and was paralleled by a similar reduction (estimated around 38% of preflight) in whole protein breakdown (Stein et al., 1999; Table 1). This indicates that a new balance between protein synthesis and degradation with a slower rate of protein turnover is achieved after prolonged exposure to microgravity. In bed rest, a transient net protein breakdown corresponds with the rapid decline in FCSA within the first 6 days of unloading, followed by a much slower decline between 6 and 57 days of unloading (Hendrickse et al., 2022; Table 2), suggesting that a new steady‐state in muscle mass was reached.

**FIGURE 1:**
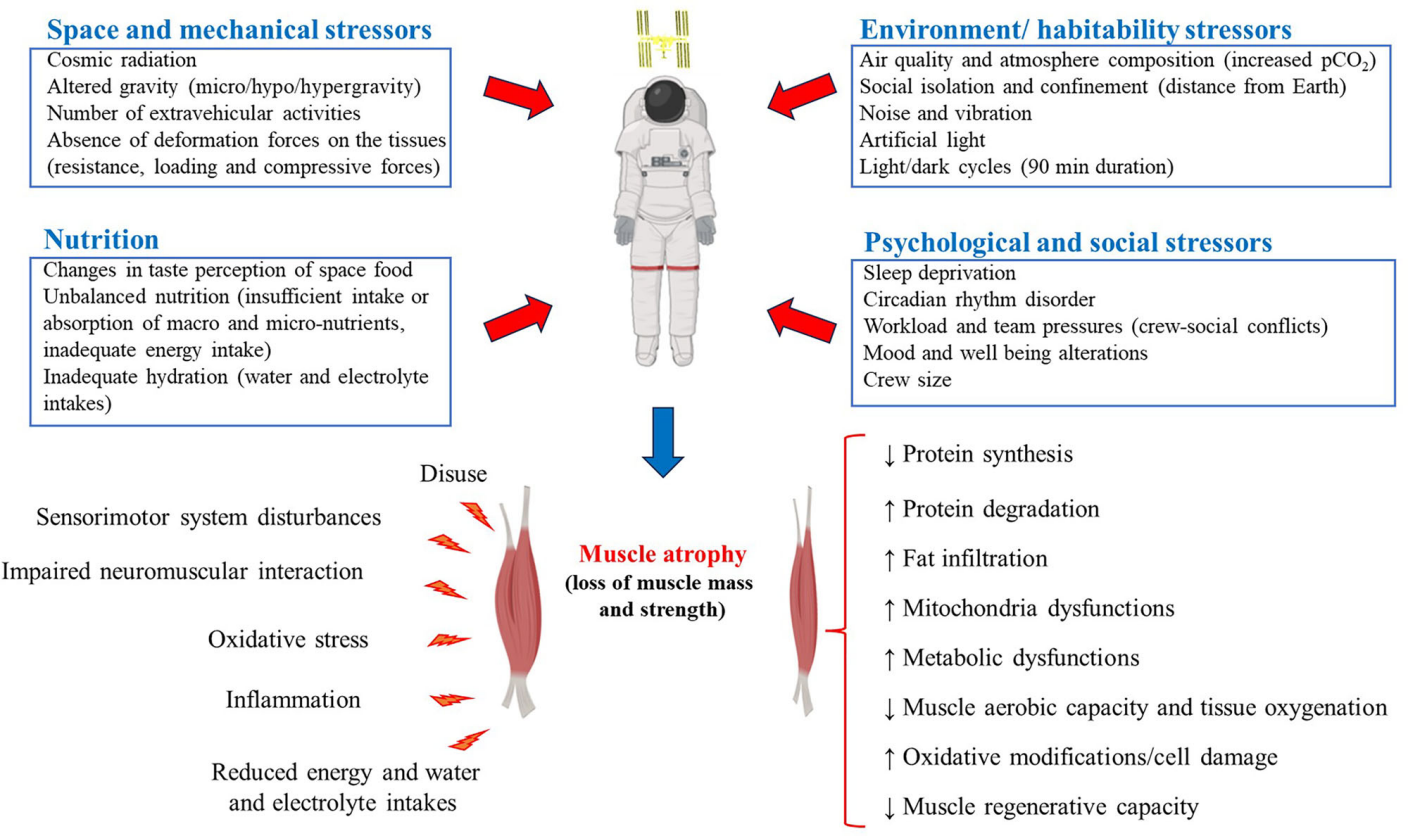
Overview of the main physiological mechanisms of spaceflight‐induced loss of muscle mass in astronauts.

At the molecular level, deactivation of Akt/mammalian target of rapamycin (mTOR) signalling is indicative of reduced protein synthesis (Chopard et al., 2009). The ATP–ubiquitin–proteasome pathway is the main proteolytic system responsible for the rapid loss of muscle proteins and the elevated expression of muscle‐specific E3 ubiquitin ligases muscle RING finger 1 (MuRF1) and muscle atrophy F‐box (MAFbx) indicates enhanced degradation of structural and myofibrillar proteins (Romanello and Sandri, 2021). Therefore, the deregulation of both pathways could contribute to muscle atrophy and loss of strength in microgravity (Figure 1). Although increased expression of MuRF1 has been depicted in atrophic myofibres following 60 days of bed rest (Salanova et al., 2008; Table 2), no significant changes were found following 21 days of bed rest (Blottner et al., 2014; Table 2). Other proteolytic pathways may also contribute to muscle atrophy and weakness in disuse/microgravity, such as the lysosomal cathepsin proteases, autophagic pathways and the Ca^2+^‐dependent non‐lysosomal cysteine proteases ‘calpains’, but their role in spaceflight‐induced muscle atrophy deserves further studies.

### 2.2 Mechanical adaptations, mitochondrial perturbation and oxidative stress in microgravity

Mechanical forces and biochemical signals, mediated by growth factors and adhesion molecules, are the central regulators of cellular functions (DuFort et al., 2011; Hudlicka et al., 1992). Cells sense mechanical stimuli that are transmitted across trans‐membrane adhesion receptors that link the cytoskeleton to the extracellular matrix and other cells (DuFort et al., 2011; Nassef et al., 2019). The activation of this matrix–integrin–cytoskeletal signalling in turn activates biochemical‐intracellular signals that govern cytoskeleton organization, cell orientation, motility, proliferation, apoptosis and growth (DuFort et al., 2011).

In vitro/in vivo (murine) studies performed during space missions, parabolic flights and microgravity created on the ground by a rotating clinostat indicated that one of the first cellular/molecular events responsible for the functional adaptation to microgravity is the perturbation in cytoskeleton organisation, which in turn leads to changes in extracellular matrix organisation, cell morphology, cell mechanical properties and the switch on/off of key signalling pathways (Hughes‐Fulford, 2003; Nassef et al., 2019). The cytoskeleton plays a central role in the spatial organisation and dynamics of cell organelles such as mitochondria and nuclei (Huang et al., 2023). For example, cytoskeletal disarrangement has been reported to lead to an aberrant mitochondrial distribution (Huang et al., 2023) that negatively affects mitochondria respiration and generation of reactive oxygen species (ROS) (Bereiter‐Hahn and Voth, 1994). In addition, cytoskeletal disarrangement may change how tension is sensed by the nuclear membrane through the Linker of the Nucleoskeleton and Cytoskeleton complex, which in turn will influence gene transcription (Burridge et al., 2019).

An impaired mitochondrial function may result in excessive ROS generation, and via local inflammation further aggravate the disuse‐induced negative protein balance, muscle wasting (Nguyen et al., 2021) and metabolic alterations (Baumann et al., 2016; Figure 1). Protein carbonylation and other oxidative modifications of myofibrillar proteins due to oxidative stress negatively impact the power‐generating capacity of muscle (Baumann et al., 2016).

Evidence of mitochondrial dysfunction in human spaceflight comes from the NASA Twins Study, where alterations in gene expression of pathways related to mitochondrial transport, mitochondrial apoptotic pathways, ROS and oxidative metabolism were found after 1 year in the twin in space compared with his homozygotic twin who stayed on the ground (Garrett‐Bakelman et al., 2019; Table 1). Moreover, proteomic, molecular and metabolomic analyses of skeletal muscle biopsies from two astronauts taken before and after a 6‐month mission onboard the ISS (the SARCOLAB pilot study) showed a reduced abundance of the mitochondrial proteome and some increases in the expression of muscle‐related stress and inflammatory factors along with diminished muscle oxidative metabolism (Capri et al., 2019, Table 1; Rittweger et al., 2018). Of note, the one astronaut who trained less vigorously, particularly regarding loading forces, showed more pronounced muscle atrophy, weakness (Rittweger et al., 2018) and pro‐inflammatory alterations (Capri et al., 2019). Similarly, the expression of autophagy regulators and ROS scavengers correlated with the level of exercise performed on board (Murgia et al., 2024; Table 1).

These observations emphasise the importance of regular high‐intensity resistance exercise as a countermeasure to attenuate microgravity‐induced decrements in muscle mass and function.

### 2.3 Muscle capillarisation

Prolonged disuse results in arterial structural remodelling and blood flow reductions to the active muscles (Thijssen et al., 2011). As shear stress and mechanical signals are crucial for the maintenance of endothelial cells, the low contractile activity in microgravity and hence few periods of high muscle blood flow will reduce stimuli that are important to maintain the capillary bed (Hudlicka et al., 1992). Yet, research on the adaptation of muscle capillarisation to microgravity is sparse. One study observed a significant reduction in the number of capillaries per fibre without alteration in the number of capillaries per unit cross‐sectional area in astronauts following 5–10 days of spaceflight (Edgerton et al., 1995; Table 1). Also, in rats the decline in FCSA was 2‐fold greater (∼−40%) than the decline in the capillary to fibre ratio (−24%) of the soleus muscle following 12.5 days of spaceflight (Desplanches et al., 1991). In both cases, this resulted in an increased, rather than reduced, capillary density.

An increased capillary density has also been observed in bed rest and denervation studies, where it has been found that capillary rarefaction may lag behind fibre atrophy resulting in an increased capillary density (Blottner et al., 2020; Hendrickse et al., 2022; Paudyal et al., 2018; Table 2). An elevated capillary density was not only found after 6 days but persisted, despite slight further atrophy, after 55 days of bed rest (Hendrickse et al., 2022). It is interesting that in studies without fibre atrophy after 19 days (Bosutti et al., 2016; Table 2), or 5 weeks (Krainski et al., 2014; Table 2) there was no evidence for capillary rarefaction in either the soleus or the vastus lateralis muscle, while following 6 weeks of bed rest, fibre atrophy was associated with a maintained capillary density in the vastus lateralis muscle (Ferretti et al., 1997; Table 2).

The capillary bed is not only important for the delivery of oxygen and substrates, such as amino acids, for protein synthesis, but also for tissue repair and growth (Hudlicka et al., 1992). Therefore, the extent of muscle capillarisation can play a crucial role in supporting muscle mass. The potential significance of the capillary bed for muscle maintenance is reflected by the close link between fibre size and the number of capillaries supplying a fibre (Bosutti et al., 2015), and the similar time course of muscle hypertrophy and angiogenesis (Plyley et al., 1998), which suggests a matching between capillary proliferation and fibre growth.

Satellite cells play a crucial role in muscle regeneration and growth. In response to damage or an anabolic stimulus, satellite cells become activated, proliferate, differentiate and fuse with each other to form new myofibres, or fuse with existing muscle fibres to repair damage or promote hypertrophy (van der Meer et al., 2011). Satellite cells are located between the sarcolemma and basement membrane of muscle fibres near capillaries, and can, therefore, easily interact with endothelial cells and elicit a coordinated angio‐myogenic response (Joanisse et al., 2017). Any changes in the co‐localisation of endothelial and satellite cells may therefore affect myogenesis and muscle tissue maintenance (Joanisse et al., 2017). The idea that these cells are mutually dependent is reinforced by the observation that the attenuated hypertrophic response and regenerative capacity in old age were associated with an increased distance between satellite cells and capillaries (Joanisse et al., 2017).

Although we observed that capillary rarefaction did not result in a reduced capillary density after 55 days of bed rest (Hendrickse et al., 2022; Table 2) it is, at least in theory, possible that capillary rarefaction may have a negative effect on muscle mass when it results in an increased distance between satellite cells and capillaries, contributing therefore to the microgravity‐induced decrease in muscle mass. This, however, needs to be further explored in spaceflight, bed rest and other models of disuse.

In summary, capillary rarefaction is proportionally less than fibre atrophy and results in improved, rather than reduced, muscle oxygenation during bed rest (Hendrickse et al., 2022; Table 2) that may facilitate any rehabilitative intervention to restore muscle mass after spaceflight.

### 2.4 Impaired neuromuscular interactions

The loss of muscle mass is, however, not the sole explanation for muscle weakness as bed rest studies often show a proportionally larger reduction in muscle strength compared to the loss of muscle volume. For example, Kawakami et al. (2001) (Table 2) reported a 10.9% reduction in knee extension force after 20 days of bed rest, but only a 7.8% decrease in muscle physiological cross‐sectional area. Similarly, Zange et al. (1997) (Table 1) observed a 20–48% decline in the maximal voluntary plantar flexion strength and only a 6–20% decrease in calf muscle volume in three cosmonauts after a 6‐month stay in the MIR space station. This disproportionally larger reduction in muscle strength than muscle size has been reported to be at least partly attributable to a reduced ability to voluntarily activate the muscles, as seen after 20 days of bed rest (Kawakami et al., 2001; Table 2).

In theory, motor neuron (MN) loss may result in a reduced ability to recruit the available muscle mass, a situation that has been reported to occur during ageing (Larsson et al., 2019). Similar to ageing, microgravity may be associated with the loss of MNs that will result in the denervation of muscle fibres. Although speculative, it has been reported that in rats 14 days of spaceflight reduces the oxidative capacity of specific groups of MNs involved in posture and locomotion, but not in those without load‐bearing functions (Ishihara et al., 2002). Therefore, one might hypothesise that over time such changes may lead to the demise of the affected MNs.

Another possibility is that impaired transmission over the neuromuscular junction (NMJ) may over time contribute to the disproportionally larger decrease in strength than muscle mass. This could be due to a loss in integrity of the NMJ, as suggested by the smaller NMJs observed particularly in the oxidative postural muscles of space‐flown rats (Deschenes et al., 2005). NMJ degradation could impair the postsynaptic signal transduction that in turn may result in an impaired force generating capacity of the remaining muscle tissue (Deschenes et al., 2005). NMJ degradation may be the result of diminished ‘muscle retrograde signals’ required for the maintenance of the NMJ, such as myokines, trophic factors and neurotrophins (Kreko‐Pierce and Eaton, 2018) and/or factors derived from MNs, such as the neural form of the proteoglycan agrin, which plays a crucial role in NMJ formation and maintenance (Sirago et al., 2023). Oxidative stress is associated with several neurodegenerative disorders, such as amyotrophic lateral sclerosis, where together with damaged mitochondria and augmented intracellular Ca^2+^ it destabilises the presynaptic membrane of the NMJ (Pollari et al., 2014). Oxidative stress in the muscle tissue could cause not only loss of NMJs but also oxidative damage via retrograde transport and ultimately loss of MNs (Pollari et al., 2014). In addition, alterations in Ca^2+^ buffering in the subsarcolemmal mitochondria located near the NMJ and a decrement in ATP production may lead to impaired neurotransmission and synapse vesicular recycling (Deschenes, 2011). A positive modulation of oxidative stress would represent, therefore, a crucial target for countermeasures directed to maintenance of skeletal muscle and NMJ stability during spaceflight.

An integral part of the NMJ is the anchorage of nicotinic acetylcholine receptors (nAChR) in the cytoskeleton through the intermediate filaments desmin and F‐actin (Mitsui et al., 2000). In in vitro (Corydon et al., 2016) models of gravitational unloading, considerable changes in the cytoskeleton have been found to be associated with desmin degradation and changes in F‐actin cytoskeletal organisation, suggesting disuse‐induced changes in the cytoskeleton may destabilise nAChR anchorage and NMJ integrity.

Despite these indications, the current literature on the main mechanisms underpinning NMJ decline in microgravity is scarce and restricted to some bed rest and space‐flown mice/rats studies. NMJ instability (reflected by an elevated concentration of serum C‐terminal agrin fragment) seems to develop just after 10 days of bed rest (Monti et al., 2021; Table 2), and in space‐flown rats, 10 days (Deschenes et al., 2005) and 21 days (Barański and Marciniak, 1990) of spaceflight caused alterations in NMJ structure that appeared to be specific (Deschenes et al., 2005) and more pronounced (Barański and Marciniak, 1990) in a postural muscle (soleus muscle). Yet, the absence of any evidence for changes in motor unit number or motor unit size in the abductor digiti minimi and tibialis anterior muscle during the 60‐day ESA–NASA AGBRESA bed rest study (Attias et al., 2020; Table 2) suggests that the loss of MNs and/or NMJ integrity is, if it occurs, of negligible magnitude. This was further supported by the absence of a significant change in circulating C‐terminal agrin fragment, a marker of NMJ damage (Ganse et al., 2021; Table 2), in the same study.

It thus appears that any changes in the NMJ and/or MN loss during spaceflight or bed rest have a minimal functional impact. However, it is possible that after longer periods of spaceflight problems with the neuromuscular interaction may develop.

### 2.5 Decline of muscle aerobic capacity and metabolic remodelling

Muscle weakness during spaceflight and bed rest is accompanied by a decrease in maximal oxygen uptake (${\dot{V}}_{O_{2}\mathrm{peak}}$) that may be mediated by a decrease in convective O_2_ transport and/or a decrease in diffusive O_2_ transport (Ade et al., 2017). Recent evidence showed that after 55 days of bed rest the fibre capillary to oxidative capacity ratio was elevated, indicating an improved, rather than decreased diffusive O_2_ transport (Hendrickse et al., 2022; Table 2). Hence, the most likely explanations for the lower whole‐body ${\dot{V}}_{O_{2}\mathrm{peak}}$ observed after bed rest or spaceflight are (i) the reduced convective O_2_ supply to the muscle as a consequence of reduced maximal cardiac output and (ii) the reduced muscle oxidative capacity (Bosutti et al., 2016; Table 2) which will result in a lower maximal O_2_ extraction (Salvadego et al., 2011; Table 2). The reduced muscle oxidative capacity and convective O_2_ supply may hamper aerobic ATP generation causing an accelerated accumulation of H^+^ and other metabolites in the muscle that, via interference with excitation‐contraction coupling and cross‐bridge cycling, may lead to an earlier onset of muscle fatigue during exercise (Degens & Jones, 2020). The reduced muscle oxidative capacity may increase reliance on glucose metabolism and result in a shift to glycolytic muscle metabolism during contractile activity after 21 days of bed rest (Bosutti et al., 2020; Table 2). The lower oxidative phosphorylation capacity and the shift to glycolysis likely contribute to the metabolic inflexibility (the flexibility to switch from fat to carbohydrate oxidation) observed after bed rest (Eggelbusch et al., 2024; Rudwill et al., 2018; Figure 1, Table 2).

It has been hypothesised that the reduction in mitochondrial function is triggered by a disuse‐induced increase in lipotoxicity and nitrosative/oxidative radical species that may ultimately lead to mitochondrial complex damage and mitochondrial removal (mitophagy) (Makrecka‐Kuka et al., 2020). In the recent 60‐day AGBRESA study we provided evidence of a temporal link between the accumulation of intracellular triglycerides, lipotoxic ceramides and sphingomyelins with a reduction in mitochondrial size, density and capacity for oxidative phosphorylation after 55 days of bed rest, yet without a significant reduction in the quality of the mitochondria (Eggelbusch et al., 2024; Table 2). Intracellular nutrient overload, as indicated by a rapid accumulation of lipids and glycogen in the muscle cells, and a reduced skeletal muscle insulin sensitivity after just 6 days of bed rest were crucial determinants of the mitochondrial alterations seen after 55 days of simulated microgravity (Eggelbusch et al., 2024; Table 2). This also suggests that a lower insulin sensitivity, at least partly caused by a decrease in GLUT‐4, precedes mitochondrial adaptations in bed rest (Eggelbusch et al., 2024; Table 2).

Surprisingly, a different finding was reported in a recent ISS experiment on five astronauts, where the expression levels of proteins involved in oxidative phosphorylation and citric acid cycle were already reduced after a short‐duration (9–11 days) mission but tended to return to normal levels after a prolonged period (>6 months) onboard the ISS (Blottner et al., 2023; Table 1). This perhaps illustrates the success of the countermeasures currently implemented in flight to restore muscle oxidative capacity.

As protein synthesis is an energy‐demanding process, a lower muscle aerobic capacity may hamper protein synthesis during spaceflight and bed rest. In this context, it is intriguing that bed rest is accompanied by a transcriptomic signature related to mitochondria, energy metabolism, and structural components that is resilient to resistance exercise countermeasures (Fernandez‐Gonzalo et al., 2020; Table 2) and perhaps explains the attenuated response to countermeasures.

Furthermore, it has been found that during ageing, impaired mito‐nuclear communication is linked to the accumulation of misfolded proteins and disrupted proteostasis (Boardman et al., 2023). In microgravity, one of the mechanisms underlying impaired mito‐nuclear communication could be elevated protein *S*‐nitrosylation (Chang et al., 2014), as seen in skeletal muscle biopsies of astronauts after >180 days onboard the ISS (Blottner et al., 2024; Table 1). In fact, these authors observed over‐nitrosylation by nitro‐DIGE analyses, after as little as 9 days of spaceflight not only in structural proteins but also in proteins involved in the TCA cycle, glycolysis, respiratory chain and creatine kinase M‐types that may impair the capacity of ATP generation (Blottner et al., 2024; Table 1). These observations emphasise the need for early interventions to prevent impairments in muscle oxidative capacity and mito‐nuclear communication.

## 3 COUNTERMEASURES TO ATTENUATE MUSCLE WASTING IN SPACE: LESSONS FROM BED REST STUDIES

Exercise elicits various adaptations in skeletal muscles, such as an increase in mitochondrial density (aerobic exercise), increases in muscle mass (resistance exercise) (Saltin & Gollnick, 1983), and improved regenerative capacity of satellite cells (NMES, endurance, resistance and high‐intensity interval training; Bazgir et al., 2016; di Filippo et al., 2017). Resistance exercise (Zunner et al., 2022) also modulates the release of endocrine factors such as inflammatory mediators, neurotrophic factors, and some myokines, such as irisin, that are critically involved in energy/glucose metabolism and muscle mass maintenance (Rai and Demontis, 2016). Aerobic exercise induces the release by the muscle of the pleiotropic cytokine interleukin‐6, which is an important link between muscle contraction and exercise‐related metabolic changes and depends on the type and duration of exercise (Fischer, 2006).

**FIGURE 2:**
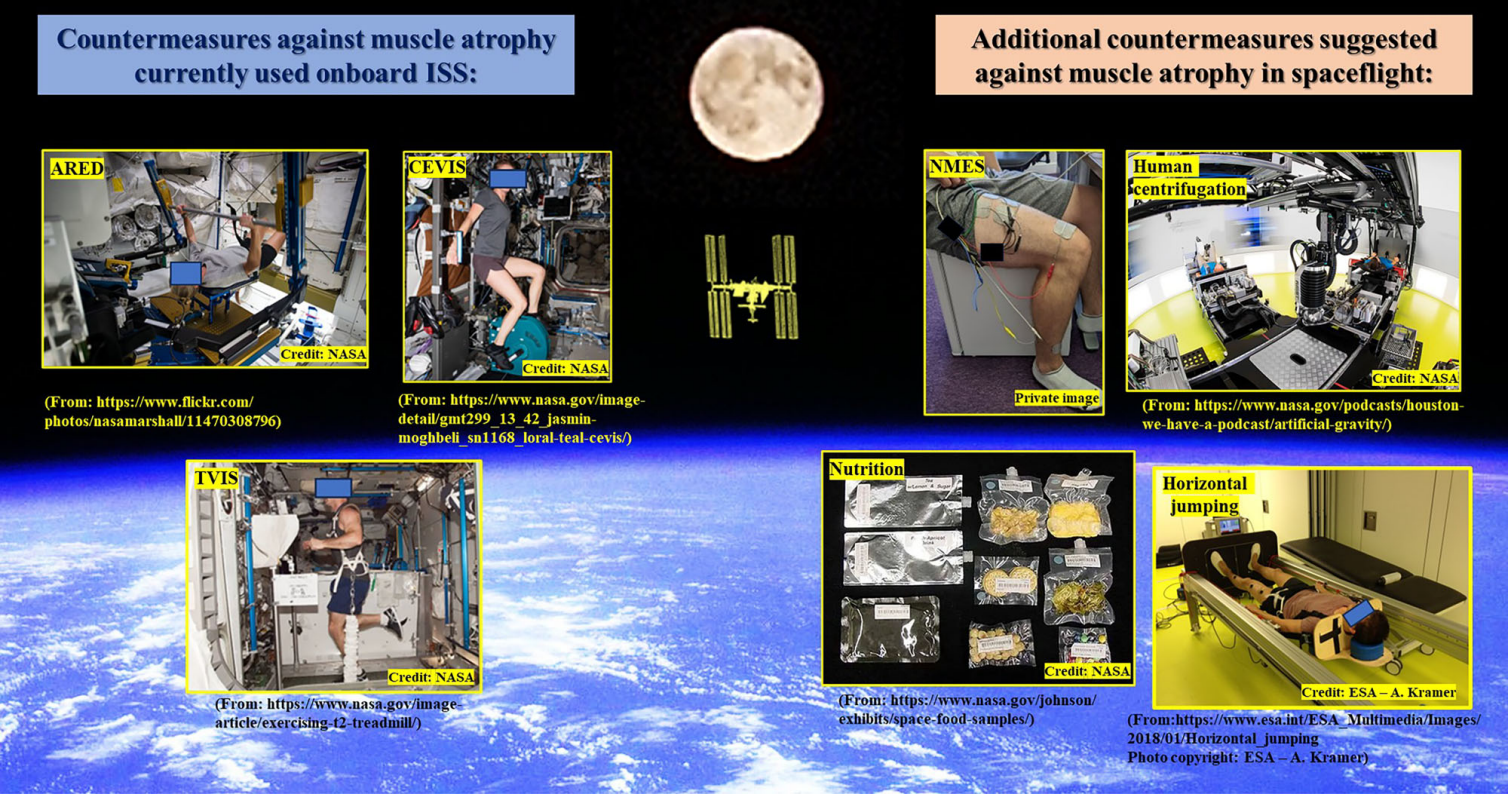
Current and novel potential countermeasures to attenuate muscle wasting in astronauts during spaceflight. Current crew members weekly exercise schedule includes the utilisation of the ARED to perform resistive exercise, the TVIS, and the CEVIS to perform aerobic exercise which attenuate aerobic and cardiovascular deconditioning. NMES exposure to AG by human centrifugation, individual nutritional countermeasures based on subject energy expenditure, exercise with increased loads (eccentric exercise training) and plyometric exercise (horizontal jumping) represent additional countermeasures suggested against muscle atrophy in spaceflight. AG, artificial gravity; ARED, Advanced Resistive Exercise Device; CEVIS, Cycle Ergometer with Vibration and Stabilization; NMES, neuromuscular electrical stimulation; TVIS, Treadmill with Vibration Isolation Stabilization System. Images: credits by NASA; credit: ESA – A. Kramer. NASA images under Creative Commons licences (https://creativecommons.org/licenses/by‐nc/2.0/).

When looking at the differences between the effects of exercise countermeasures, supine cycling (aerobic exercise) attenuated the reduction in aerobic capacity and orthostatic tolerance in bedrest (Shibata et al., 2010; Table 2), while resistance exercise (strength training) attenuated the reductions in muscle mass and strength (Akima et al., 2003; Table 2) so effectively that both are now applied on the ISS (Petersen et al., 2016). Vibration superimposed to resistive exercise appeared to improve the effectiveness of resistive exercise to preserve muscle size, strength and energy metabolism (Kenny et al., 2017; Table 2), as well as vasodilation of small arterioles that would improve the delivery of O_2_ and nutrients to exercising muscle and the removal of carbon dioxide and metabolites (Beijer et al., 2015) during bed rest. Of all the exercise countermeasures, reactive jumps (Figure 2) appeared to be the most promising exercise countermeasures tested in prolonged bed rest (60 days; Thomasius et al., 2026). Based on plyometric training principles, reactive jumps were so effective for maintaining muscle power and strength, bone mass in the lower extremities, and providing cardiovascular benefits that they have been considered as a potential exercise modality to be applied in space (Thomasius et al., 2026). Onboard the ISS, currently each ESA crew member is assigned to a daily exercise time slot of 2.5 h that includes a combination of sessions on three available exercise devices to attenuate microgravity‐induced adaptations. Currently, the equipment includes the Advanced Resistive Exercise Device (ARED), the Treadmill with Vibration Isolation Stabilization System (TVIS) and the Cycle Ergometer with Vibration and Stabilization (CEVIS). While resistance training with the ARED helps to attenuate the loss of muscle mass, aerobic exercise on the TVIS and CEVIS helps to counteract cardiovascular deconditioning (Petersen et al., 2016; Figure 2).

In addition to exercise countermeasures, nutritional supplements and anti‐osteoporotic drugs have shown effectiveness in bed rest and may be implemented in long‐term spaceflight. For example, during a previous ESA bed rest study, it was found that whey protein supplementation with potassium bicarbonate effectively attenuated the reduction in muscle mitochondrial oxidative capacity and ${\dot{V}}_{O_{2}\max}$ during 21 days of bed rest (Bosutti et al., 2016; Table 2), which confirmed the potential benefit of adequate dietary supplementation on muscle metabolism and function in microgravity. Nutrient cocktail supplementation based on a mix of a bioactive polyphenol compound and omega‐3 fatty acids showed a potential protective effect against oxidative damage in muscle during 60 days of bed rest, but no effects in preventing/mitigating the loss of muscle strength and muscle mass (Arc‐Chagnaud et al., 2020; Table 2). Others have recommended an integrated countermeasure consisting of nutrition (amino acid/protein supplementation) and exercise to maintain muscle mass during prolonged bed rest (Blottner et al., 2014; Table 2; Figure 2). Nonetheless, the combination of whey protein/bicarbonate supplementation with resistance vibration exercise showed just a marginal effect, alleviating ${\dot{V}}_{O_{2}\max}$ reduction but not the cardiovascular deconditioning induced by 21 days of bed rest (Guinet et al., 2020; Table 2).

When it comes to nutritional interventions, particular attention should be directed to the exact daily energy expenditure of astronauts (Stein et al., 1996), as astronauts during prolonged space missions could encounter severe negative energy balance, due to a lower dietary intake (due to loss of appetite), increased energy expenditure during extravehicular activities, and the intense exercise regimes that they usually follow during the mission. Despite the well‐balanced currently available space diet (Pittia et al., 2023), the astronaut energy intake is typically ∼25% below the energy requirement, and this could have a negative impact on protein turnover and enhance muscle atrophy (Pittia et al., 2023; Stein et al., 1996). It is therefore important to consider the additional energy and protein requirement of the exercise programme and ensure appropriate intake.

## 4 AG AND CENTRIFUGATION INTERVENTIONS

**FIGURE 3:**
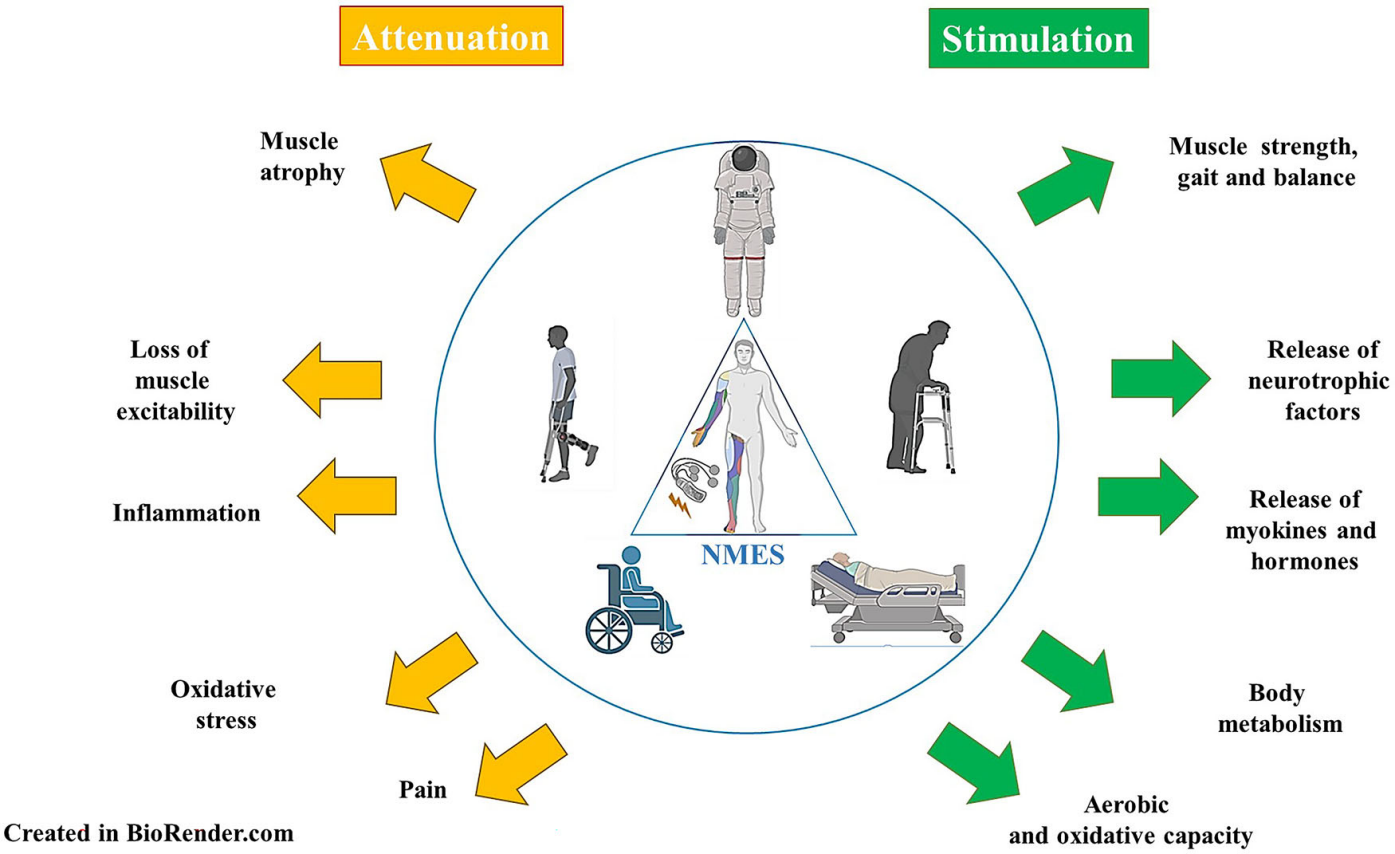
Overview of the main physiological responses activated by neuromuscular electrical stimulation (NMES) to attenuate muscle wasting in space and disuse conditions.

Despite the considerable advancements in exercise programmes to prevent microgravity‐induced deconditioning in spaceflight, in most astronauts, complete maintenance of muscle mass in microgravity is still not achieved. The principal cause of the limited effectiveness of existing exercise countermeasures is the difficulty in achieving high enough exercise loads in space (Rittweger et al., 2018). The development of countermeasures that can impose Earth‐like loading forces may help to counteract space‐flight‐induced muscle atrophy. This is one of the reasons for the growing interest in AG as a countermeasure of microgravity‐induced physiological adaptations. The rationale is that AG, generated by a short‐arm centrifuge, mimics via centrifugal forces, our natural 1‐G environment. By creating a gravitational gradient along the major body axis, AG has the potential to prevent multiple aspects of long‐term exposure to weightlessness, including fluid shifts, cardiac shrinkage and loss of bone and muscle mass (Figure 3). In support of this, it has been found that after 21 days of bed rest, 1 h a day of AG (2.5 G at the feet) superimposed to bed rest showed a maintained rate of protein synthesis (Symons et al., 2009; Table 2) without significant muscle atrophy and a net gain in the torque–velocity relationship of the knee extensor and plantar flexor muscle groups (Caiozzo et al., 2009; Table 2). These early results were, however, not corroborated by the 60‐day bed rest ‘AGBRESA’ study (Clément et al., 2022; Table 2), where neither daily 30 min intermittent nor continuous AG (1 G at the heart) attenuated the bed‐rest‐induced muscle fibre atrophy (Hendrickse et al., 2022; Table 2) or loss of muscle strength (Kramer et al., 2021; Table 2). Although we have no explanation for the discrepancy between these studies, it has been suggested that it is necessary to combine AG with exercise to act as an effective countermeasure for microgravity‐induced decrements in cardiovascular and neuromuscular function (Hargens et al., 2013). Even if AG enhances the benefit of resistance exercise, it poses a significant challenge to application in space due to the size of the equipment. Indeed, future long‐term missions such as flights to asteroids, moons or planets such as Mars, in *cis*‐lunar orbit (Lunar Gateway) and presently to the space station necessitate drastic payload restrictions and require efficient, light and small countermeasure devices.

Any countermeasure must at the same time have no adverse health risks, and adequately warrant effectiveness and safety. In addition, such countermeasures need to provide a functional return to a terrestrial environment and promote an optimal rate of post‐flight recovery. In Tables 1 and 2 are listed the key space‐flight and bed rest studies discussed in the present review.

## 5 NMES

An intervention that is applied with light and small devices, and is safe and effective over a large range of stimulation frequencies is NMES (Crevenna et al., 2003). During NMES, trains of electrical stimuli are delivered by a portable pulse generator via electrodes to superficial muscles to elicit sub‐maximal contractions. NMES has been shown to not only counteract muscle atrophy following prolonged periods of disuse, immobilisation, injury, or in patients affected by chronic debilitating conditions (Gibson et al., 1988; Jones et al., 2016; Maffiuletti et al., 2019; Zange et al., 2017), but also to improve motor output, gait, balance, muscle function (Paillard, 2018) and whole‐body aerobic capacity (Crevenna et al., 2003; Figure 2).

Overall, NMES can be considered as an exercise mimetic, since the adaptations in skeletal muscles are similar to those in response to conventional exercise, such as increases in muscle strength and endurance, fibre mitochondrial content and a glycolytic‐to‐oxidative shift in the metabolic profile of muscle fibres (Akagi et al., 2023; Cabric et al., 1988; Gondin et al., 2011). Also, the mediators of these adaptations are similar to those involved in the typical exercise response, including the release of hormones and endocrine factors such as myokines (Figure 3), inflammatory cytokines and activation of various intracellular signalling pathways (Gibson et al., 1988). For example, NMES delivered with 8‐ to 50‐Hz trains was effective and non‐strenuous to enhance both the strength and endurance capacity of the skeletal muscles and to improve ${\dot{V}}_{O_{2}\mathrm{peak}}$, endothelium‐independent vasodilation and walk distance and decrease circulating levels of ROS and pro‐inflammatory cytokines in patients with severe chronic heart failure (Dobšák et al., 2012). High frequency (>50 Hz) NMES has been found to increase skeletal muscle regenerative capacity of satellite cells while reducing oxidative stress in healthy older people (di Filippo et al., 2017) as well as to reduce myostatin and increase MyoD gene expression 2–4 h after its cessation in older people with type 2 diabetes (Wall et al., 2012). Proteomic analysis showed that 8 weeks of isometric 75‐Hz NMES (25 sessions of 18 min) resulted in a fast‐to‐slow phenotype transition that was accompanied by a metabolic shift from glycolytic to oxidative metabolism and improved the antioxidant defence systems in healthy subjects (Gondin et al., 2011; Figure 3).

The efficacy of NMES to increase or attenuate deconditioning‐induced decreases in muscle mass is proportional to the amount of force/tension generated by the stimulation (Gondin et al., 2011). It has been suggested that the intensity of the elicited contraction, also referred to as NMES training intensity, is the most important determinant of the effectiveness of NMES for the maintenance of muscle mass and strength (Maffiuletti et al., 2019). Perhaps this proportionality is related to the fact that during NMES only part of the muscle is activated and the recruited muscle mass increases with increasing current (Adams et al., 1993).

Besides NMES training intensity, the electrically evoked force–frequency relation indicates that the force during NMES is not only dependent on the muscle mass recruited but also on stimulation frequency. Indeed, animal studies suggest that the stimulation frequency shows a dose–response relationship with the elicited muscular adaptations (Sutherland et al., 1998), which could also be the case in humans. While a high force is desirable, the beneficial effects of high‐force contractions may be limited by the rapid onset of muscle fatigue (Maffiuletti et al., 2014). It thus appears important to consider the settings of the NMES parameters such as current amplitude, pulse duration, pulse frequency, duty cycle, ramp time, pulse pattern and programme duration to determine the nature (strength vs. endurance) and efficiency of the desired adaptations. Yet, so far there are no systematic studies that assess the trade‐off between high‐intensity NMES and fatigue for the desired benefits (particularly for the application of NMES during a disuse period), and this explains the need for testing different NMES protocols in different study conditions, including spaceflight (Figure 2).

Furthermore, NMES in conjunction with nutrition supplements, such as dietary protein supplements, might enhance sustaining muscle mass and strength; however, the evidence is too limited for a final conclusion (Maffiuletti et al., 2019). Novel stimulation systems allow the simultaneous stimulation of several muscle groups in different areas of the body. The simultaneous stimulation of multiple muscles is commonly called ‘whole‐body neuromuscular electrical stimulation’ (WB‐NMES). This technology is based on the application of a wearable suit and has been found to effectively induce a metabolic response, and when combined with aerobic or voluntary exercises, to enhance glucose metabolism (Watanabe et al., 2019). Nevertheless, because data are still scarce, particularly in the disuse context, and some risks have been evidenced (Stöllberger and Finsterer, 2019), it is too premature to draw firm conclusions concerning the efficacy of WB‐NMES to combat muscle wasting in disuse conditions.

Overall, NMES is a promising tool to attenuate spaceflight‐induced muscle wasting. NMES, although it has been applied successfully in clinical settings for a number of decades (Maffiuletti et al., 2023), has not yet found its way to routine application in hospitals, for example, on intensive care wards, let alone space travel. This is potentially due to the fact that muscle atrophy per se does not increase the risk of mortality in young and healthy populations and is reversible in an acceptable timespan (LeBlanc et al., 2000). NASA, however, currently considers muscle atrophy amongst the five highest‐priority human health risks for a mission to Mars (Patel et al., 2020). Despite promising effects observed on earth, NMES enhancement of the onboard exercise protocols has hitherto not been systematically investigated. Nonetheless, some pilot studies suggest that NMES is feasible on board the ISS and potentially helps to prevent muscle atrophy in upper and lower limb muscles (a detailed list of studies is reported elsewhere; Maffiuletti et al., 2019). NMES therefore deserves to be tested for its in‐flight feasibility. Table 3 lists the key NMES training studies and their major outcomes discussed in this section.

**TABLE 3:** Summary of key NMES training studies and major outcomes.

Authors	Population	Condition	Stimulation pattern	Outcome vs. controls
Crevenna et al. (2003)	59 years	Patients with heart failure and implanted pacemakers	20 NMES sessions; NMES combined protocol at different frequencies (8–50 Hz): 60 s at 8 Hz, 4 s at 15 Hz, 4 s at 30 Hz and 6 s at 50 Hz to knee extensor and flexor muscles	NMES appeared safe even in patients with heart failure and pacemakers
Jones et al. (2016)	53–71 years, M = 54%	Patients with advanced chronic respiratory disease, chronic heart failure, cancer or HIV/AIDS	Stimulation parameters range of: stimulation frequency 50 (15–75) Hz, pulse duration 400 (200–700) µs, target duty cycle 33 (13–75)%, session length 30 (18–240) min, session frequency 5 (2–7) times each week, and programme duration 6 (4–11) weeks	NMES appeared effective for treating (quadriceps) muscle weakness in adults with advanced progressive disease
Zange et al. (2017)	26.4 ± 3.7 years, M	58 days’ unloaded calf musculature by orthotic device	NMES + lupin protein supplementation. 30 Hz, 5 s ON/ 5 s OFF for 20 min 2×/day	Reduced loss of volume in triceps surae muscle. Reduced loss of plantar flexion torque
Cabric et al. (1988)	Young men	Healthy students	19 days NMES in gastrocnemius muscle. NMES protocol: high frequency and current amplitude	Increased FCSA type II fibres, mitochondrial fraction, nuclear volume and nuclear DNA content
Gondin et al. (2011)	26 ± 3 years, M	Healthy volunteers	25 sessions of bilateral isometric NMES of the quadriceps muscle over 8 weeks. 75 Hz with a rise time of 1.5 s, a steady tetanic stimulation time of 4 s, and a fall time of 0.75 s (total duration of the contraction: 6.25 s). Warmup: 5 min 5 Hz	Increased maximum voluntary force and neural activation. Fast‐to‐slow and glycolytic‐to‐oxidative metabolic shift; strengthened cytoskeleton; enhanced intracellular anti‐oxidant defence
Akagi et al. (2023)	22 ± 2 years, M	Healthy subjects	8‐week NMES training for 3 days/week. 2‐min warm‐up, 9‐min main training programmes containing five different NMES programmes at varying frequencies (12–60 Hz), and 2‐min cooling down	Increased muscle strength, muscle hypertrophy of the knee extensor muscles
Dobšák et al. (2012)	59 years	Patients with chronic heart failure	12‐week NMES of quadriceps and calf muscles. 10 Hz, 20 s ON/20 s OFF, 60 min 2x/day	Improved arterial stiffness and stabilised autonomic balance. Increased ${\dot{V}}_{O_{2}\mathrm{peak}}$
Di Filippo et al. (2017)	69.5 ± 1.6 years, M	Healthy elderly	40 NMES sessions over 8‐weeks. 75 Hz with a rise time of 1.5 s, a plateau of 4 s, and a fall of 0.75 s	Improved muscle strength and mobility. Improved regenerative capacity of skeletal muscle
Wall et al. (2012)	70 ± 2 years	Patients with type 2 diabetes	60 Hz, biphasic rectangular waveform electrical current with pulse duration of 500 µs. 1 session of 60 min NMES	Stimulated skeletal muscle protein synthesis rates
Watanabe et al. (2019)	20.7 ± 0.9 years, M	Healthy volunteers	40 Hz, whole body NMES + aerobic exercise	Combination WB‐NMES and aerobic exercise enhanced the metabolic response to a level equivalent to high intensity exercise

NMES, neuromuscular electrical stimulation; WB‐NMES, whole‐body NMES.

## 6 CONCLUSIONS

Several countermeasures have been developed over the last few decades to combat muscle wasting and sustain astronaut performance and health while off‐Earth. Although exercise training is the most effective countermeasure to mitigate neuromuscular and cardiovascular deconditioning in microgravity, for most astronauts the current onboard exercise programmes and devices do not offer complete protection. NMES represents a training modality which could potentially enhance exercise‐induced improvements in muscle form and function. As it is delivered with a small and lightweight type of ‘exercise equipment’, it could even be used in future space missions and reduced gravity habitats with no room for voluminous equipment.

Muscle atrophy and the loss of resistance to fatigue are common effects of immobilisation in patients with chronic and acute diseases and the elderly. Sarcopenia is the main component of frailty and loss of independence in old age. It is therefore highly important for all these populations to develop and test new countermeasures and treatment options. The study of the effects of NMES under microgravity conditions (in space or bed rest) will not only improve the countermeasures program available for future space travels but also serve as a basis for useful applications in the clinical setting and to counteract the effects of ageing on muscle function in the elderly population.

## AUTHOR CONTRIBUTIONS

Study design: Alessandra Bosutti and Hans Degens. Writing—original draft preparation: Alessandra Bosutti and Hans Degens. Writing and final editing: Alessandra Bosutti and Hans Degens All authors contributed to writing‐review and editing the manuscript. All authors have read and approved the final version of this manuscript and agree to be accountable for all aspects of the work in ensuring that questions related to the accuracy or integrity of any part of the work are appropriately investigated and resolved. All persons designated as authors qualify for authorship, and all those who qualify for authorship are listed.

## CONFLICT OF INTEREST

None declared.

## Data Availability

The data that support the findings of this study are available from the corresponding author upon reasonable request.
